# Effects of Social Support and Loneliness on the Irrational Consumption Tendencies of Healthcare Products among the Elderly: A Structural Equation Model

**DOI:** 10.3390/ijerph192114404

**Published:** 2022-11-03

**Authors:** Yating Chen, Luqi Li, Zhiji Tan, Chengcheng Ma, Binyan Wang, Qing Guo, Li Li

**Affiliations:** 1 School of Public Health, Hangzhou Normal University, Hangzhou 310036, China; 2 School of Humanity and Management, Zhejiang Chinese Medical University, Hangzhou 310053, China; 3 Library, Zhejiang University of Technology, Hangzhou 310014, China; 4 School of Public Health, Zhejiang Chinese Medical University, Hangzhou 310053, China; 5 School of Law, Zhejiang University City College, Hangzhou 310015, China

**Keywords:** social support, loneliness, irrational consumption tendencies, the elderly

## Abstract

Background: In recent years, with the development of the social economy and an improvement in health consciousness, the levels of demand and consumption for healthcare products have been increasing rapidly among the elderly. However, the irrational consumption of healthcare products has caused widespread concern, as it can generate economic losses and have negative effects on psychological health. Therefore, it is critical to identify the variables that can reduce tendencies toward irrational consumption in the area of healthcare products. This study aimed to explore the relationship between the irrational consumption tendencies of healthcare products, social support, and loneliness among elderly people in Hangzhou, China. Methods: In 2021, a cross-sectional survey involving 485 elderly people from three districts in Hangzhou, China, was conducted. Descriptive statistics were calculated for socioeconomic status and demographic characteristics, level of loneliness, social support, and irrational consumption tendencies. A structural equation model was used to test the impact of social support on the irrational consumption tendencies of healthcare products among the elderly and to explore the mediating effects of loneliness. Results: The findings reveal that the average levels of social support and loneliness among the elderly were 30.63 points (total = 44 points) and 6.88 points (15 points), respectively. The average scores of the four subscales of irrational consumption tendencies, which were named susceptibility to persuasion, interpersonal influences, pursuit of added value, and fears of aging were 2.48, 2.93, 2.48, and 3.17 points (5 points), respectively. Social support had a significant effect on irrational consumption tendencies, and loneliness played a partial mediating role between social support and irrational consumption tendencies. Conclusions: A relationship model was constructed to examine the association between loneliness, social support, and irrational consumption tendencies among older people in relation to healthcare products. From a social support and psychological perspective, advice and countermeasures are proposed to prevent the irrational consumption of healthcare products among older people.

## 1. Introduction

China’s aging society has had a significant impact on consumption, and the country’s large elderly population has become an important consumer group of many products on the current market. With the development of the social economy and the increase in the living standards of Chinese residents, health awareness among the elderly is constantly enhanced, which is manifested in the increasing consumption and even irrational consumption of healthcare products [1,2,3]. Irrational consumption can be defined as the irrational purchase decision made by consumers when they are affected by various factors. It can also be characterized by impulsiveness (i.e., unconsidered purchase behavior or sudden purchase intention) and blindness (i.e., purchase intention caused by following trends or being influenced by the outside world) [4]. The phenomenon of the irrational consumption of healthcare products among the elderly often occurs against the backdrop of a multitude of other factors, such as the physiological and psychological factors that impact older people, health knowledge reserves, family structure, levels of social interaction, and the marketing of healthcare products [2,5,6,7]. Older people buying healthcare products irrationally and taking them unscientifically not only causes serious financial loss and increased financial burden but also has adverse effects on the physical and mental health of older people [8]. In recent years, the irrational consumption of healthcare products by the elderly has caused widespread concern in society [2,9]. It is very important to take measures to prevent the elderly, who have irrational consumption tendencies, from developing irrational consumption behaviors. In this study, the irrational consumption tendency of healthcare products among the elderly refers to the tendency or the willingness to make an irrational purchase decision on healthcare products among the elderly.

At present, neither China nor the international community has provided a clear definition of healthcare products [10]. In mainland China, the General Standard for Health (Functional) Food GB16740-97, defines healthcare products as being comparable to general food types, as they are able to regulate the functions of the human body. They are regarded as suitable for certain people to eat but are not recognized as being able to cure diseases [11]. In foreign countries, these products are called dietary supplements (nutraceuticals), which are generally regarded as supplements that offer health benefits extending beyond their basic nutritional value, since they not only supplement an individual’s diet but also contribute to the prophylaxis and/or treatment of a disease or disorder [12].

Due to excessive propaganda of healthcare products and the absence of adequate health education, older people have a lack of knowledge about healthcare products. Meanwhile, the sellers of healthcare products use means such as celebrity publicity and online marketing to publicize the information about healthcare products falsely, thus misleading the consumption choices of the elderly and increasing the possibility of irrational consumption of healthcare products [13]. A survey that was carried out to examine healthcare product awareness among the elderly in Chongqing, China, showed that 23.42% of participants viewed healthcare products as a panacea. Moreover, only 14.13% and 28.7% of the elderly paid attention to the batch numbers and labels on healthcare products during their purchasing process. In China, 48.1% of the elderly did not know the production batch numbers of the healthcare products at all, and 18.8% mistakenly believed that healthcare products could cure diseases [14]. The studies show that most elderly have a lack of knowledge about healthcare products and misunderstand the function of healthcare products [15].

A vague concept of healthcare products has led to confusion and misunderstanding when elderly people purchase healthcare products. For example, elderly people have doubts about the following questions: Are healthcare products a substitute for food? Are healthcare products equivalent to drugs? Is the dosage of healthcare products not limited? Should healthcare products be applicable to a specific group? Is the quality of healthcare products directly proportional to the price and exquisite packaging [16,17,18,19]?

By analyzing the related literature, the irrational consumption of healthcare products is often defined as the consumption behaviors, such as purchasing useful but unsuitable healthcare products, paying money that is beyond the scope of one’s consumption ability, purchasing healthcare products that are unapproved or even harmful to one’s health. According to the Survey Report on the Consumption of Healthcare Products for the Elderly released by the Shanghai Consumer Rights and Interests Protection Commission in 2018, nearly 70% of older people had purchased healthcare products in the last year, and nearly 40% of children believed that their parents had an irrational consumption of healthcare products [20,21].

Because most elderly have a lack of knowledge about healthcare products, there are irrational consumption behaviors without self-awareness. The study focused on the irrational consumption tendencies of healthcare products among the elderly.

Studies related to the consumption of healthcare products have referred to consumption motivations, influencing factors of consumption, customer fraud, and irrational consumption. The irrational consumption of healthcare products among the elderly is affected by many factors [22,23]. In an investigation into the consumption of healthcare products among 400 elderly people in Qingdao and Yantai, the results suggested that the factors affecting purchasing decisions mainly included physical health, psychological factors, cognitive function, recommendations by acquaintances, and advertising [24].

Due to a decline in physical functioning and greater susceptibility to chronic diseases, health-related needs play an important role in irrational consumption among the elderly [25]. In addition, because the health literacy level of the elderly is generally low, there is no sufficient knowledge to support correct selections for health products [26,27,28]. Psychologically, seeking mental comfort and finding ways of minimizing loneliness are also significant factors that influence decisions to purchase healthcare products among the elderly [25]. There are different views on the relationship between cognitive function and irrational consumption among the elderly. Some studies have shown that age-related cognitive decline leads to reliance on simpler strategies. That is to say that older adults tended to look up less information and take longer to process it and used simpler, less cognitively demanding strategies [29]. During medical decision-making, older people have higher error rates in processing information than other populations, regardless if the task simple or complex [30]. However, a few studies have found a rich experience and strong information processing ability of the elderly in completing decision-making tasks [31]. One particular survey about consumer behavior in relation to healthcare products found that, among older people, the emotional value and emotional experience were positively correlated with trust and purchasing intentions [3]. Moreover, in terms of familial and social factors, family support, social support, and participation in social activities may influence the consumption of healthcare products in older people [9]. By reviewing the extant literature, the factors influencing irrational consumption were identified and can be summarized in the three dimensions of physiological factors, psychological factors, and social factors. Although physiological factors, such as cognition, comprehension, and memory decline, may be important motivations for irrational consumption among the elderly [32], social factors (e.g., social support) and psychological factors (e.g., loneliness) are also important predictors.

Social support refers to the general or specific supportive resources that individuals can gain from others or from their social networks, which can help them to deal with certain difficulties [33,34]. A total of 40% of older people in China live alone, which can lead to serious social isolation [26,27,28]. With advancing age, retirement, illness, and fewer opportunities to participate in society, older people are limited in the range of activities that they can engage in. The elderly are more likely to be restricted in their ability to access information, which may hinder their ability to utilize positive social supports [35,36,37,38].

The ability to access and process information tends to be hampered by insufficient social support, which may increase the likelihood of irrational consumption. However, another study showed that excessive social interaction may promote consumption by comparison [32]. When faced with an absence of information, individuals are more likely to show high levels of interpersonal trust [39,40]. Older adults rely more on interpersonal influence than deliberation when making choices [41]. They may be especially susceptible to emotional advertising appeals and marketing campaigns that do not provide useful information or are deceptive, thus increasing the likelihood that older adults will fall victim to scams [42].

These findings suggest that there may be a mediating variable between social support and irrational consumption. Several studies have shown that social support is related to loneliness [2,43]. Loneliness is a feeling of a gap between the interpersonal relationship that an individual has and the interpersonal relationship that they expect to have. Weiss divided loneliness into emotional loneliness and social loneliness [44]. Loneliness is largely borne out of being isolated from the outside world, and it can be alleviated by strengthening social connections with others [28]. The main factors affecting loneliness in the elderly are demographic factors and social support. Low levels of contact with family and friends and low levels of activity are uniquely linked to social loneliness [45,46].

Some studies have shown that social support has positive effects on reducing loneliness in the elderly [2]. To reduce the negative impact of loneliness and insufficient social support, older people should actively engage in wider social interaction with groups or organizations so as to enjoy better social support [25].

The mental demand of some older adults may be satisfied by the spiritual comfort gained from the social network [47,48]. However, there may be different ways that elderly people seek social support, and they may also experience varying intensities of loneliness, which may lead to differences in irrational consumption tendencies [32].

According to the psychological characteristics of the elderly, this study aimed to examine the relationship between social support and the irrational consumption tendencies of healthcare products, while also exploring the mediating effect of loneliness.

## 2. Materials and Methods

### 2.1. Design and Sample

A cross-sectional study was conducted from 1 May to 30 June 2021, in Hangzhou, Zhejiang Province, China. The sample size calculation formula was as follows: $n=z_{1-\alpha ∕2}^{2}Ρ\left(1-Ρ\right)∕\mu^{2},{\;z}_{1-\alpha ∕2}^{2}$ = 1.96, μ = 0.05. According to data sourced from the Zhejiang Statistics Yearbook, during this period, Hangzhou was home to 1,882,618 older people. We assumed Ρ = 0.5, and based on the formula calculation, n = 384. Considering the possible sample loss, a pre-survey was conducted in Binjiang District, Hangzhou, and 50 questionnaires were distributed, of which 41 questionnaires were deemed valid. Finally, the sample was increased by 18%, such that n = 468.

To enhance the representativeness of the sample, this study employed a stratified multistage sampling design. First, based on per capita GDP, Hangzhou was divided into high, medium, and low GDP groups (Gongshu District, Xihu District, and Qiantang District). According to the sample size and resident population data of Hangzhou, out of the 468 samples, 147 (31.4%), 157 (37.4%), and 146 (31.2%) samples were from the Gongshu District, Xihu District, and Qiantang District, respectively. Second, two streets were randomly selected from each district. Third, two communities were randomly selected from each street. Home, community activity centers, and community health centers were randomly selected in each community, considering differences in health status and social participation among older adults. As in the National Population Census of China, both the people in the age group of 60 and over and the people in the group of 65 and over were all counted as senior citizens. In this study, the minimum age standard for participants was 60 years old.

The inclusion criteria for this study were as follows: (1) Nationality of the People’s Republic of China; (2) age ≥ 60 years; (3) voluntary participation in this study; (4) participators can complete the questionnaire by themselves or with the help of investigators; (5) participants can understand the meaning of each item in the questionnaire. Exclusion criteria included: (1) those with inconvenient movement, confusion, or mental disorders; (2) people who are unwilling to cooperate.

### 2.2. Data Collection

The research team members received standardized training before the survey, and distributed the questionnaires to elderly people in the selected communities. The questionnaire included a cover page explaining the purposes and procedures of the study. The respondents were assured that participation in the survey was voluntary, and the return of questionnaires represented informed consent. The questionnaires with missing data or obvious contradictions in logic were excluded. Valid questionnaires were sorted and numbered. A total of 500 questionnaires were distributed, and 485 valid questionnaires were retrieved. The effective recovery rate was 97%.

### 2.3. Measures and Variables

The survey questionnaire was composed of four sections, and the Cronbach’s alpha value was 0.848.

Section 1 of the questionnaire focused on the basic characteristics of the respondents, including gender (male, female), age (60–69, 70–79, 80–89, ≥90 years old), pre-retirement occupation (laborers and farmers, enterprise staff, science/education/culture/health, cadres/freelancers/other), education level (primary school and below; middle school; high school or technical school; junior college; or undergraduate and above), medical insurance (without medical insurance; rural and urban resident basic medical insurance; commercial medical insurance; urban employee basic medical insurance), health status (no chronic diseases; one chronic disease; two or more diseases).

Section 2 assessed the level of loneliness. Referring to the previous literature that considered methods of measuring loneliness, loneliness was comprehensively assessed in relation to three factors in this study: friendship, social interaction, and belonging. The respondents were requested to rate how often they felt that they did not have enough friends, were lonely, and did not belong to a social group, based on a five-point Likert scale (1 = never, 2 = rarely, 3 = sometimes, 4 = often, 5 = always). The total score was 15 points, and higher scores were taken to indicate higher levels of loneliness. The Kaiser–Meyer–Olkin (KMO) value was 0.687.

Section 3 assessed social support, which was measured by the self-made social support scale, based on the Social Support Rating Scale (SSRS) developed by Xiaoshuiyuan in 1986. According to the results of the pre-survey, and combined with the theme, the self-made social support scale includes three dimensions, namely subjective support, objective support, and support utilization, and a total of seven items. The score of each item was consistent with the SSRS. For items 1–5, the score of each item ranged from 1–4; for item 6, respondents stated no source (0) or have sources (scores ranged from 1–8, depending on the number of sources; and item 7 was scored according to the sum of four items. The total score of the self-made social support scale was 44, and higher scores indicated higher levels of social support. The KMO values of the subjective support subscale, objective support subscale, and support utilization subscale were 0.733, 0.726, and 0.683, respectively.

Section 4 measured irrational consumption tendencies using a self-made evaluation scale, which included 13 items. Then factor analysis, which is not discussed in this paper, yielded four subscales that comprised 12 items. The four-subscale solution accounted for 68.768% of the overall variance. These four subscales of irrational consumption tendencies were named susceptibility to persuasion, interpersonal influences, pursuit of added value, and fears of aging. They individually accounted for 23.395%, 17.736%, 14.324%, and 13.312% of the overall variance, respectively. Each item was scored on a scale ranging from 1 to 5, and the total score on the scale was 60. Higher scores indicated stronger irrational consumption tendencies among the respondents. The KMO value was 0.728.

### 2.4. Data Analysis

In this study, SPSS V.19.0 (IBM, New York, NY, USA) and AMOS 26.0 (IBM, New York, NY, USA) were used to analyze the survey results. First, the basic characteristics of the respondents were shown by frequency (N) and percentage (%). Second, we calculated the statistical data, including the means (M) and standard deviations (SD) to show the scores of loneliness, social support, and irrational consumption tendencies among the respondents. Finally, structural equation models were employed to verify the path and synthetic relationship between social support, loneliness, and irrational consumption tendencies among older adults. Statistical significance was set at *p* < 0.05.

## 3. Results

### 3.1. Basic Characteristics of Respondents

Table 1 shows that 50.93% of the respondents were female, and most respondents were aged 60–69 years old (55.05%). Prior to retirement, the majority of respondents had worked as enterprise employees, laborers, and farmers (55.8%). Most respondents had a primary school education (40%) and middle school education (44%). Fifty-four percent of respondents held medical insurance for rural and urban residents, while more than 80% reported that they suffered from more than one chronic disease.

### 3.2. Level of Social Support for Older People

Table 2 shows that the social support score of respondents was 30.63 ± 4.87. The scores were mainly concentrated around 25–30 points (35.46%) and 30–35 points (37.73%).

### 3.3. Level of Loneliness among Older People

The results of Table 3 show that the average score for loneliness among the elderly in Hangzhou was 6.88, with a minimum score of 3 and a maximum score of 14. Fifty-nine percent of older adults had a total loneliness score of 5 to 10, 34% had a total loneliness score of less than 5, and only 7% had a total loneliness score of more than 10.

Using the statistically determined value of 27% as the critical point [49], the *t*-test revealed a significant difference between the upper 27% and lower 27% of the loneliness score, which was divided into three categories: high, medium, and low levels of loneliness.

### 3.4. Levels of Irrational Consumption Tendencies

Levels of irrational consumption tendencies were reported in Table 4. The fear of aging (3.17 ± 0.93) subscale of irrational consumption tendencies rated the highest, followed by interpersonal influences (2.93 ± 1.01), susceptibility to persuasion (2.48 ± 0.88), and pursuit of added value (2.48 ± 0.84).

### 3.5. Path Analysis of Social Support, Loneliness, and Irrational Consumption Tendencies

**Figure 1:**
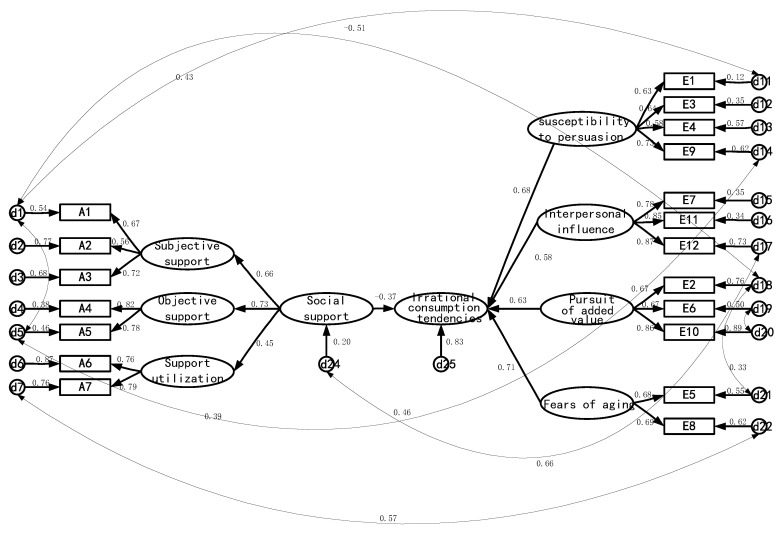
Structural equation model of social support and irrational consumption tendencies.

Figure 1 shows the structural equation model of social support and irrational consumption tendencies among the elderly with regard to healthcare products. The results show that social support had a significant impact on the irrational consumption tendencies among the elderly in Hangzhou as far as healthcare products were concerned (γ = −0.37, *p* < 0.001).

**Figure 2:**
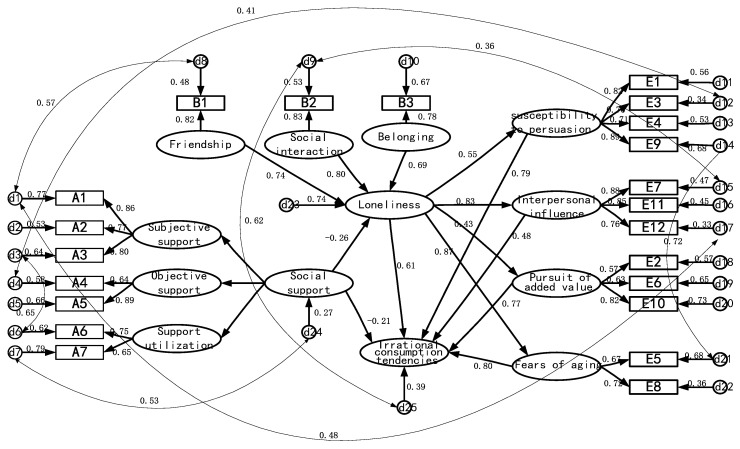
Structural equation model of social support, loneliness, and irrational consumption tendencies.

Figure 2 presents the structural equation model with solitary mediating variables. The results show that social support had a significant effect on irrational consumption tendencies (γ = −0.21, *p* < 0.001), loneliness had a significant effect on irrational consumption tendencies (γ = 0.61, *p* < 0.001), and the consumption effect of social support and loneliness was significant (γ = −0.26, *p* < 0.05), indicating that loneliness played a partial mediating role in social support and irrational consumption tendencies, such that the mediating effect of loneliness accounted for 43%.

## 4. Discussion

### 4.1. The Effects of Loneliness and Social Support on Irrational Consumption Tendencies

The path analysis (Figure 1) shows a negative relationship between social support and the irrational consumption tendencies of healthcare products among older people (γ = −0.37). Similarly, previous studies related to social support have found that the effective use of social support can help the elderly to obtain external support so as to reduce the tendency of irrational consumption [48,50]. On the one hand, studies have shown that social support can reduce the fear of aging in older adults [51], and fear of aging is an important factor in irrational consumption tendency. On the other hand, the elderly with high social support are more likely to obtain effective information and have a stronger ability to identify information, thus reducing the tendency of irrational consumption [2,9].

The path analysis (Figure 2) also shows a positive regulatory relationship between loneliness and irrational consumption tendencies (γ = 0.61). Higher levels of loneliness were associated with higher irrational consumption tendencies of healthcare products. This is in line with James’s findings [50]. The average value of irrational consumption propensity was significantly higher in the high-loneliness group than in the low-loneliness group. In addition, the latent variable loneliness had a potential regulating effect on interpersonal influences (γ = 0.83) and fears of aging (γ = 0.87) as observed variables. A study showed that loneliness affects attitudes toward aging and perceived health in older people [52]. The pursuit of health is an important motive for the irrational consumption of healthcare products [2,12]. Another study showed that the consumption of healthcare products can satisfy the needs of the elderly for establishing interpersonal relationships and provide them with psychological comfort [25].

Social support was negatively correlated with loneliness (γ = −0.26). Acierno et al. also examined the association between social support and mental health, suggesting that low social support significantly increased the risk of virtually all forms of mistreatment among the elderly, and then harmed mental health [23], which is similar to our research. The latent variable loneliness had a potential regulating effect on interpersonal influences (γ = 0.83) and fears of aging (γ = 0.87). Through social support, loneliness may play a partial mediating role in regulating irrational consumption tendencies regarding healthcare products. Some scholars have divided loneliness into social loneliness and emotional loneliness, and by increasing social support, social loneliness can be reduced, thus reducing the irrational consumption tendency of the elderly [2,53].

### 4.2. The Principal Components of Irrational Consumption Tendencies

The factor loading scores of four subscales are also shown in Figure 2. The fear of aging (γ = 0.80), a susceptibility to persuasion (γ = 0.79), and a pursuit of added value (γ = 0.77) ranked in the top three positions for irrational consumption tendencies, while interpersonal influences (γ = 0.48) were in the lowest position among the four principal components.

The fear of aging describes the perceptions of older people who regard themselves as tired and worn out; many lose their sense of self-worth, and with advancing age and the onset of chronic disease, these sentiments can intensify. A previous study showed that advancing age and chronic disease may lead to a strong sense of aging and a loss of dignity [54]. In addition, a study suggested that health-conscious individuals tended to more strongly espouse a prevention-oriented attitude than those who were less health conscious [55]. A recent study showed that health literacy had the most significant effects on consumption because people with high health literacy can access and study information and make appropriate decisions regarding selection and consumption [56].

Regarding self-worth, a study showed that there was a significant negative relationship between self-worth and death anxiety [57]. Episodic memory decline became faster after retirement among English civil servants, but decline in other cognitive domains did not accelerate [58]. Moreover, people with higher self-worth usually experience more positive emotions than negative ones, and their satisfaction with life also increases, leading to an improvement in subjective well-being [59].

However, mental aging and physical aging are not synchronized; as such, helping older adults to face physical changes and finding ways to alleviate any negative emotions that they may have can help to reduce irrational consumption tendencies [2,9,25,60].

A susceptibility to persuasion refers to the fact that older people are susceptible to the influence of authority figures or others. Usually, the elderly are resistant to unfamiliar products, and follow habits or obey authority when they make consumption decisions [42,61]. This is different from our research in that our foothold was to focus on external influences, while some studies focus on the elderly’s own habits. Many elderly people cannot clearly identify the effects of healthcare products because of their insufficient health knowledge [62]. In the marketing activities of healthcare products, some businesses use a variety of means to falsely publicize inferior healthcare products, and others unilaterally exaggerate the positive role of healthcare products on health, which lead the vulnerable elderly to make irrational consumption decisions [42,63,64,65]. In addition, some studies have shown that public opinion is important for public healthcare product education [66,67]. Public opinion should be rationally used to improve the perception of the consumption of healthcare products for the elderly [68].

The pursuit of added value refers to ancillary products, which may include concessions and gifts offered by merchants, or the sense of superiority and satisfaction that results from having obtained valuable products. In Li’s research, status, price, and prestige were shown to be factors influencing consumer purchasing behavior [69]. Sellers of healthcare products often exploit these psychological characteristics of the elderly in their marketing campaigns [12]. The elderly people who have poor judgment and self-control are easily seduced and attracted by the added value [69].

## 5. Conclusions

The current study found that loneliness and social support had effects on irrational consumption tendencies. Social support had a significant effect on irrational consumption tendencies, and loneliness played a partial mediating role between social support and irrational consumption tendencies. Social support was negatively related to loneliness. Fears of aging, susceptibility to persuasion, and the pursuit of added value were principal components of irrational consumption tendencies of healthcare products. These results suggest that we should improve social support to reduce loneliness and thus reduce irrational consumption tendencies among the elderly.

## Figures and Tables

**Table 1:** Basic characteristics of respondents in this study.

Characteristics		Number (N)	Percentage (%)
Gender	Male	238	49.07%
	Female	247	50.93%
Age	60–70	267	55.05%
	70–80	134	27.62%
	80–90	75	15.46%
	≥90	9	1.85%
Pre-retirement occupation	Laborers and farmers	169	34.85%
	Enterprise staff	125	25.77%
	Science/education/culture/health	78	16.08%
	Cadres	77	15.88%
	Freelance	22	4.54%
	Other	14	2.89%
Education level	Primary school and below	195	40.21%
	Middle high school or technical school	214	44.12%
	Junior college or undergraduate and above	76	15.67%
Medical insurance	Without medical insurance	14	2.89%
	Rural and urban residents’ basic medical insurance	262	54.02%
	Commercial medical insurance	107	22.06%
	Urban employee medical Insurance	102	21.03%
Health status	No chronic diseases	45	13.40%
	Have one chronic disease	172	42.68%
	Have two or more chronic diseases	190	43.92%

**Table 2:** Social support scores of older people in Hangzhou.

Social Support Scores	Number (N)	Percentage (%)	M ± SD
≤20	13	2.68%	30.63 ± 4.87
20–25	44	9.07%	
25–30	172	35.46%	
30–35	183	37.73%	
35–40	67	13.81%	
≥40	6	1.25%	

**Table 3:** Confirmatory *t*-test for stratification of the loneliness level.

Group	Number (N)	M ± SD	SE	T	*p*	95% CI
Loneliness scored in the lower 27%	131	4.61 ± 1.50	0.16	−31.15	0.000	−7.12~−6.27
Loneliness scored in the upper 27%	131	11.31 ± 1.35	0.14			

**Table 4:** Scores of influencing factors for irrational consumption tendencies.

Factors of Irrational Consumption Tendencies		M ± SD
Susceptibility to persuasion		2.48 ± 0.88
	Susceptibility to persuasion	
	Lack of information	
	Blindly follow	
	Obedience to authority	
Interpersonal influence		2.93 ± 1.01
	Introversion	
	Focus on the senses	
	Spiritual comfort	
Pursuit of added value		2.48 ± 0.84
	Covet interests	
	Gaining a sense of superiority	
	Satisfaction of desire	
Fears of aging		3.17 ± 0.93
	Health concerns	
	Fear of death	

## Data Availability

Data are available from the authors at reasonable written request after authorization by the Data Protection Office of Zhejiang Chinese Medical University, China.
